# Transcriptome profiling of lung immune responses potentially related to acute respiratory distress syndrome in forest musk deer

**DOI:** 10.1186/s12864-022-08917-7

**Published:** 2022-10-11

**Authors:** Jie Tang, Lijuan Suo, Feiran Li, Kun Bian, Chao Yang, Yan Wang

**Affiliations:** 1 Shaanxi Institute of Zoology, Xi’an710032, Shaanxi, China; 2 Shaanxi Provincial Field Observation & Research Station for Golden Monkey, Giant Panda and Biodiversity, Xi’an 723400, Shaanxi, China

**Keywords:** Forest musk deer, RNA sequencing, Lung, Immunity, Bacterium, Virus, Fungus

## Abstract

**Background:**

Forest musk deer is an endangered species globally. The death of captive forest musk deer can be caused by certain respiratory system diseases. Acute respiratory distress syndrome (ARDS) is a huge threat to the life of forest muck deer that breed in our department.

**Methods:**

Lung histopathologic analysis was conducted by hematoxylin and eosin (HE) staining. The lung gene changes triggered by ARDS were examined by RNA sequencing and related bioinformatics analysis in forest musk deer. The potential functions of unigenes were investigated by NR, SwissProt KOG, GO, and KEGG annotation analyses. Vital biological processes or pathways in ARDS were examined by GO and KEGG enrichment analyses.

**Results:**

A total of 3265 unigenes were differentially expressed (|log_2_fold-change|> 2 and adjusted *P value* < 0.01) in lung tissues of 3 forest musk deer with ARDS compared with normal lung tissues of the non-ARDS group. These differentially expressed unigenes (DEGs) played crucial roles in immunity and defense responses to pathogens. Moreover, we identified the DEGs related to one or more of the following biological processes: lung development, immunity, and bacterial/viral/fungal infection. And six DEGs that might be involved in lung injury caused by immune dysregulation or viral/fungal infection were identified.

**Conclusion:**

ARDS-mediated lung gene alterations were identified in forest musk deer. Moreover, multiple genes involved in lung development and lung defense responses to bacteria/viruses/fungi in ARDS were filtered out in forest musk deer.

**Supplementary Information:**

The online version contains supplementary material available at 10.1186/s12864-022-08917-7.

## Introduction

Forest musk deer (*Moschus berezovskii*), mainly distributed in China and Vietnam, are listed as “Critically Endangered under criterion A2cd” in the International Union for Conservation of Nature (IUCN) RED LIST CATEGORY AND CRITERIA (version 3.1) [1]. Moreover, forest musk deer are classified as a first-class nationally-protected wildlife species under the Chinese Wild Animal Protection Law [2, 3]. Forest musk deer can secrete the musk, which has multiple potential pharmacological properties such as neuroprotective, antioxidative, anti-inflammatory, and anticancer effects in the traditional Asian medicine industry [4, 5]. Moreover, musk has been widely used in the international perfume industry [6, 7]. Thus, many forest musk deer were hunted and trapped in the past, which resulted in a steep decline in the population of forest musk deer.

To relieve the poaching and resource pressure on wild forest musk deer, the Chinese government has encouraged enterprises to establish artificial breeding systems for forest musk deer since the 1950s [8]. Despite much progress in the artificial breeding of forest musk deer in China, the long-term development of forest musk deer breeding is hindered by multiple diseases, including respiratory disorders [9–11]. In the animal farming process, acute respiratory distress syndrome (ARDS) is a major threat to the life of captive forest musk deer. ARDS, a form of acute respiratory failure with a higher mortality rate, is closely linked with multiple diseases including acute lung injury, sepsis, and pneumonia [12–14]. Moreover, it has been reported that infections with multiple pathogens (*e.g.,* bacteria, viruses, fungi, and parasites) and dysfunctions of the immune system are involved in the development of pneumonia, acute lung injury, and ARDS [14–16]. In-depth insight into the molecular mechanisms underlying ARDS might deepen our understanding of the pathogenesis of ARDS and contribute to the better management of ARDS.

In this project, ARDS-induced lung gene alterations were examined by RNA sequencing (RNA-seq) in forest musk deer. Moreover, the major biological processes or signaling pathways regulated by these differentially expressed unigenes (DEGs) were identified. Additionally, multiple DEGs that might play vital roles in lung development, immunity, and infections of pathogens (*e.g.,* bacteria, viruses, and fungi) were screened out.

## Materials and methods

### Animals and sample collection

The animal experiments were performed with the approval of the Ethics Committee of Shaanxi Institute of Zoology. Forest musk deer were raised in the Shaanxi Institute of Zoology (Xi an, China). Lung samples were collected from 3 adult forest musk deer (2♂1♀, 4.5 years old) with ARDS that died within 3 days of the onset of respiratory symptoms and 3 adult forest musk deer(♂, 3.5 years old) that died from fighting against each other. The middle lobe samples of the right lungs were collected immediately after death in the control group. Two anterior lobe samples and one middle lobe sample of the right lungs were obtained within 4 h after natural death in the ARDS group. Lung tissue samples were stored at -80 °C.

### Histopathologic analysis

The histopathologic alterations of lung tissues were examined by hematoxylin and eosin (HE) staining. Briefly, paraffin-embedded lung tissue Sects. (4 μm) were prepared after fixation, dehydration, and vitrification. Next, the lung tissue sections were treated and stained using the Hematoxylin and Eosin Staining Kit (Beyotime, Shanghai, China) following the protocols of the manufacturer.

### RNA extraction and library preparation

Total RNA was extracted from diseased lung tissues (Pne_1, Pne_2, and Pne_3) (ARDS group) and normal lung tissues (Ctrl_1, Ctrl_2, and Ctrl_3) (no-ARDS group) using the Trizol reagent (Thermo Scientific, Waltham, MA, USA) according to the protocols of the manufacturer. RNA concentration and purity were examined by NanoDrop 2000 spectrophotometer (Thermo Scientific). RNA purity and integrity were tested by gel electrophoresis on a 1% agarose gel. Also, the concentration and integrity of RNA were further analyzed using the Agilent RNA Nano 6000 Assay Kit on Agilent Bioanalyzer 2100 (Agilent Technologies, Santa Clara, CA, USA). Next, high-quality RNA (RNA Integrity number > 7.0; 1 µg/sample) was used in the RNA sample preparations. The cDNA libraries were constructed using the NEBNextUltra RNA Library Prep Kit for Illumina (NEB, Ipswich, MA, USA) following the manufacturer’s instructions and index codes were added. Briefly, mRNA with poly-A structure was enriched using poly-T oligo-attached magnetic beads and then fragmented using divalent cations under elevated temperature in NEBNext First Strand Synthesis Reaction Buffer (5X). Then, mRNA was reversely transcribed into first-strand cDNA using M-MuLV Reverse Transcriptase (RNase H) and random hexamer primer. Next, second-strand cDNA was synthesized using DNA Polymerase I and RNase H. Subsequently, remaining overhangs of cDNA were converted into blunt ends and NEBNext Adaptor with hairpin loop structure were ligated with adenylated DNAs (3’ends). The cDNA library fragments (250 ~ 300 bp) were purified with the AMPure XP system (Beckman Coulter, Brea, CA, USA). Next, cDNA was co-incubated with USER Enzyme (NEB) for 15 min at 37 °C. After enzyme inactivation for 5 min at 95 °C, the PCR reaction was performed using Phusion High-Fidelity DNA polymerase, Universal PCR primers, and Index Primer. Next, PCR products were purified using the AMPure XP system (Beckman Coulter), and the quality of the cDNA library was evaluated on the Agilent Bioanalyzer 2100 system (Agilent Technologies). The index-coded samples were clustered on a cBot Cluster Generation System using TruSeq PE Cluster Kit v3-cBot-HS (Illumia) according to the manufacturer’s instructions. After cluster analysis, the libraries were sequenced on the Illumina Novaseq platform with paired-end 150 bp reads.

### Data analysis

The quality of raw data was evaluated using the FASTQCv0.10.1 software (parameters: extract/f fastq/t 18). Reads with less than or equal to 2 mismatches against the primers/adaptors and low-quality reads with more than 10% non-ATCG (unknown) bases or 50% of bases with the Q score ≤ 5 were removed using the NGS QC TOOLKIT v2.3.3software as previously described [17–19]. Also, the statistical information of raw reads/bases, clean reads/bases, valid bases (clean reads/raw reads *100%), Q20, Q30, and GC content was collected. The reference genome sequences of forest musk deer were downloaded from the ENA database (https://www.ebi.ac.uk/ena/browser/view/PRJNA317652?show=reads). Next, clean reads after quality control were aligned to the reference genome of forest musk deer using the Hisat2v2.1.0 software(parameters: fr/dta/ufflinks/S) and assembled into unigenes using the StringTiev1.3.3b software (default parameters).

### Unigene annotation

The annotation analysis of unigenes was performed by comparison against the NCBI non-redundant (NR), SwissProt, clusters of orthologous groups for eukaryotic complete genomes (KOG), gene ontology (GO), and Kyoto encyclopedia of genes and genomes (KEGG) databases using the Basic Local Alignment Search Tool (BLAST) algorithm [20]. Forest musk deer unigenes with significant sequence homology to the genes/proteins in these databases (E value < 1E-5) were obtained and the annotation information was retrieved. GO and KEGG annotation analysis was performed using the Blast2GO software or KAAS online tool, respectively. Blast2GO and KAAS can be used for the automatic functional annotation of DNA or protein sequence data in non-model species with no available annotation information [21–23].

### Differential expression, enrichment, and Venn analysis

Differential expression analysis was performed using the DESeq2 R package. The ARDS versus non-ARDS model was used in the DEseq2 analysis. Unigenes with |log_2_fold change|> 2 and adjusted *P value* < 0.01 were identified to be differentially expressed. Additionally, GO and KEGG enrichment analyses were conducted and the significance was examined by a hypergeometric distribution test. Terms with a *P value* < 0.05 were considered to be significantly enriched. Venn analysis was used to compare lists by jvenn online tool (http://jvenn.toulouse.inra.fr/app/example.html), which can display the specific or shared elements of different lists.

## Results

### Anatomical and histopathologic analysis of lung tissues of forest musk deer

**Fig. 1:**
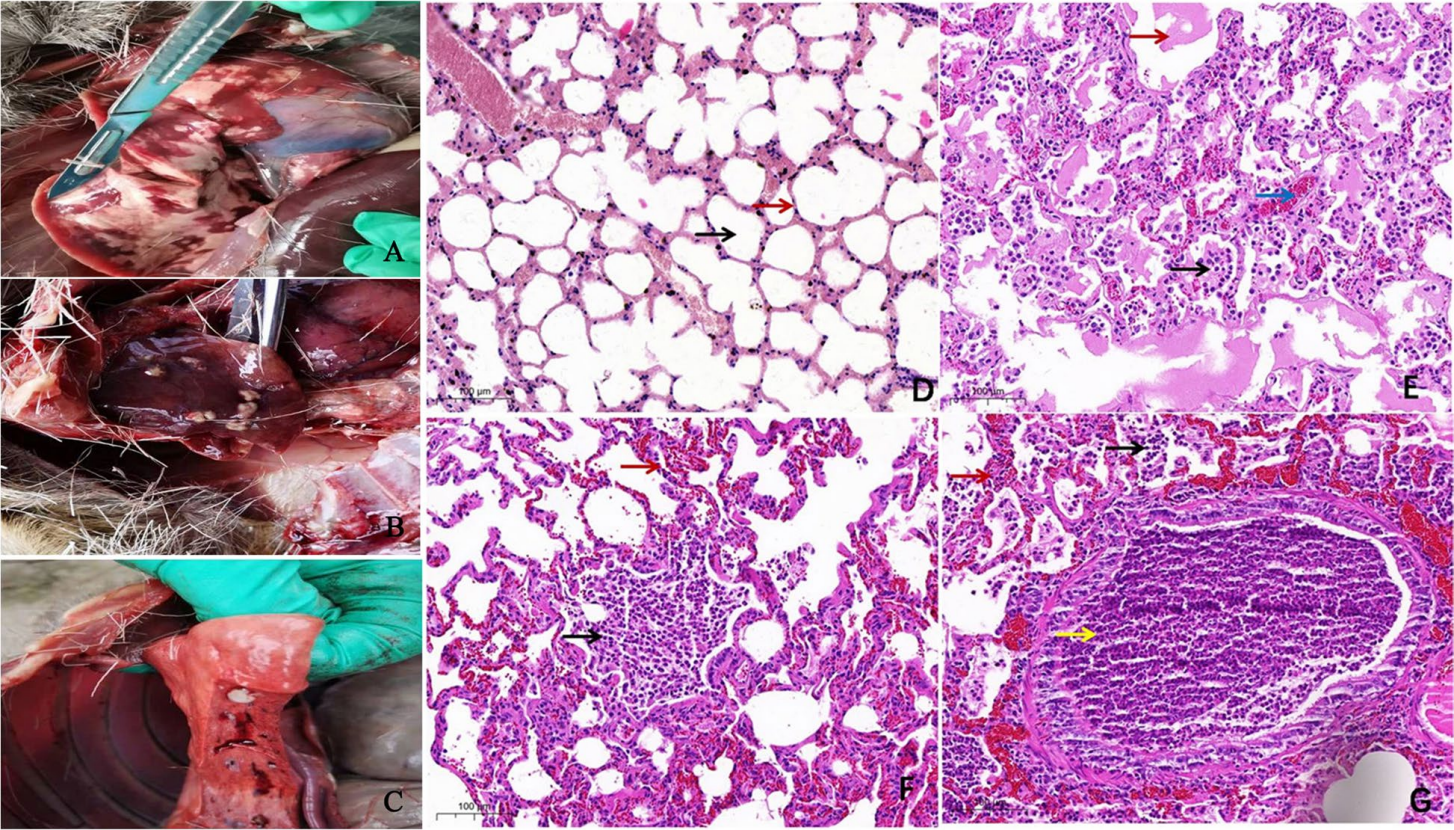
Anatomical and histopathologic analysis of forest musk deer lung tissues. (**A-C**) The clinical-pathological alterations of lung tissues of 3 forest musk deer that died of ARDS. (**D**) HE staining image of normal lung tissue. Black arrow: alveoli; Red arrow: alveolar wall. (**E-G**) HE staining images of lung tissues of forest musk deer with ARDS. The black arrow in Fig. 1**E**-1**G** represents the pus cells in the alveolar space. The red arrow in Fig. 1**E** indicates the exudates in the alveolar space. The red arrow in Fig. 1**F** denotes the alveolar wall capillary congestion. The red arrow in Fig. 1**G** indicates pulmonary interstitial congestion. The yellow arrow in Fig. 1**G** indicates a large number of pus cells in the bronchi

As presented in Fig. 1A-C, the lung tissues of forest musk deer with ARDS presented notable clinical-pathological alterations such as purulence and hemorrhage. HE staining of normal lung tissue shows normal alveoli and alveolar walls in Fig. 1D. Histopathologic analysis showed that there were copious pus cells (Fig. 1E-G) and exudates (Fig. 1E) in the alveolar cavity and noticeable congestion in the pulmonary interstitium (Fig. 1G) and alveolar wall (Fig. 1E) in the lung tissues of forest musk deer with ARDS. Moreover, the bronchi were filled with pus cells in the ARDS group (Fig. 1G).

### Illumina sequencing and assembly

**Fig. 2:**
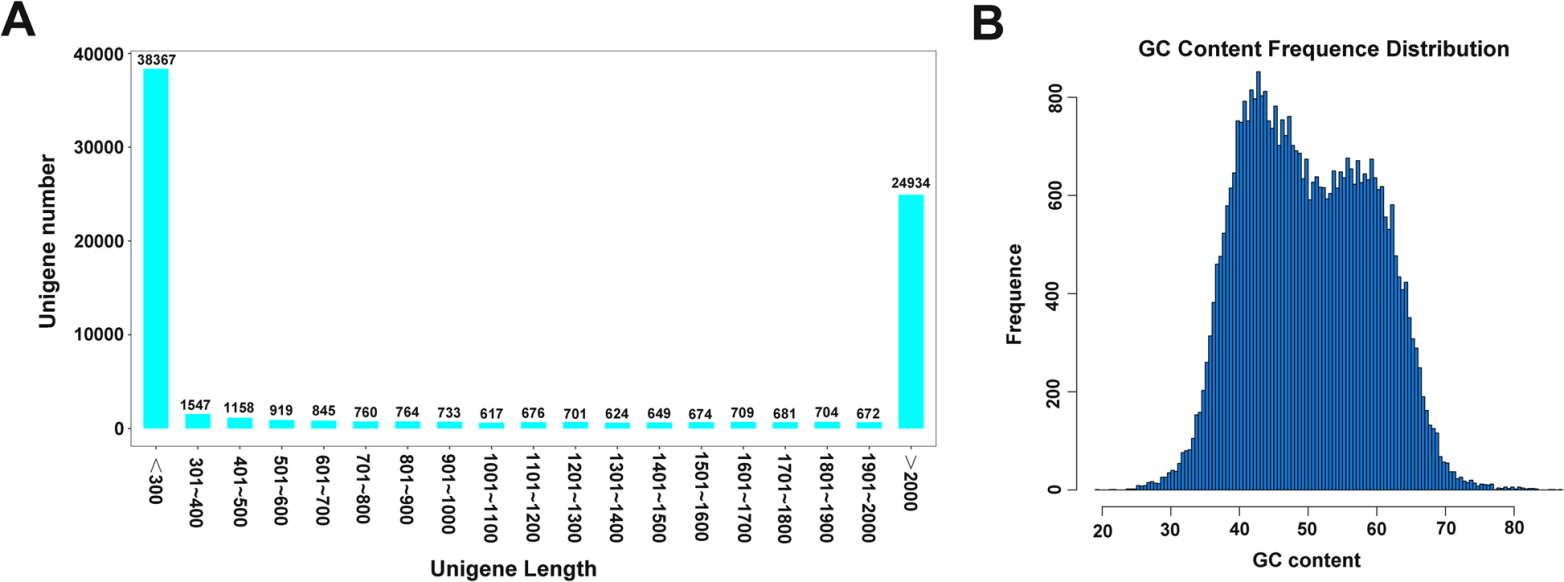
Statistical analysis of unigenes. (**A**) Length distribution patterns ofunigenes. (**B**) GC content frequency distribution patterns of unigenes

To identify genes that might play crucial roles in ARDS-induced lung injury, the lung gene changes triggered by ARDS were investigated by RNA-seq in forest musk deer. As shown in Table S1, the Q30 values (percentage of bases with the sequencing error rate of less than 0.1%) were more than 94%, and the GC percentages were approximately 50%, suggesting the production of high-quality sequencing data. Next, the clean reads were assembled de novo into unigenes using StringTie software. The length and GC content distribution patterns of unigenes are presented in Fig. 2A and B.

### Functional annotation analysis

**Fig. 3:**
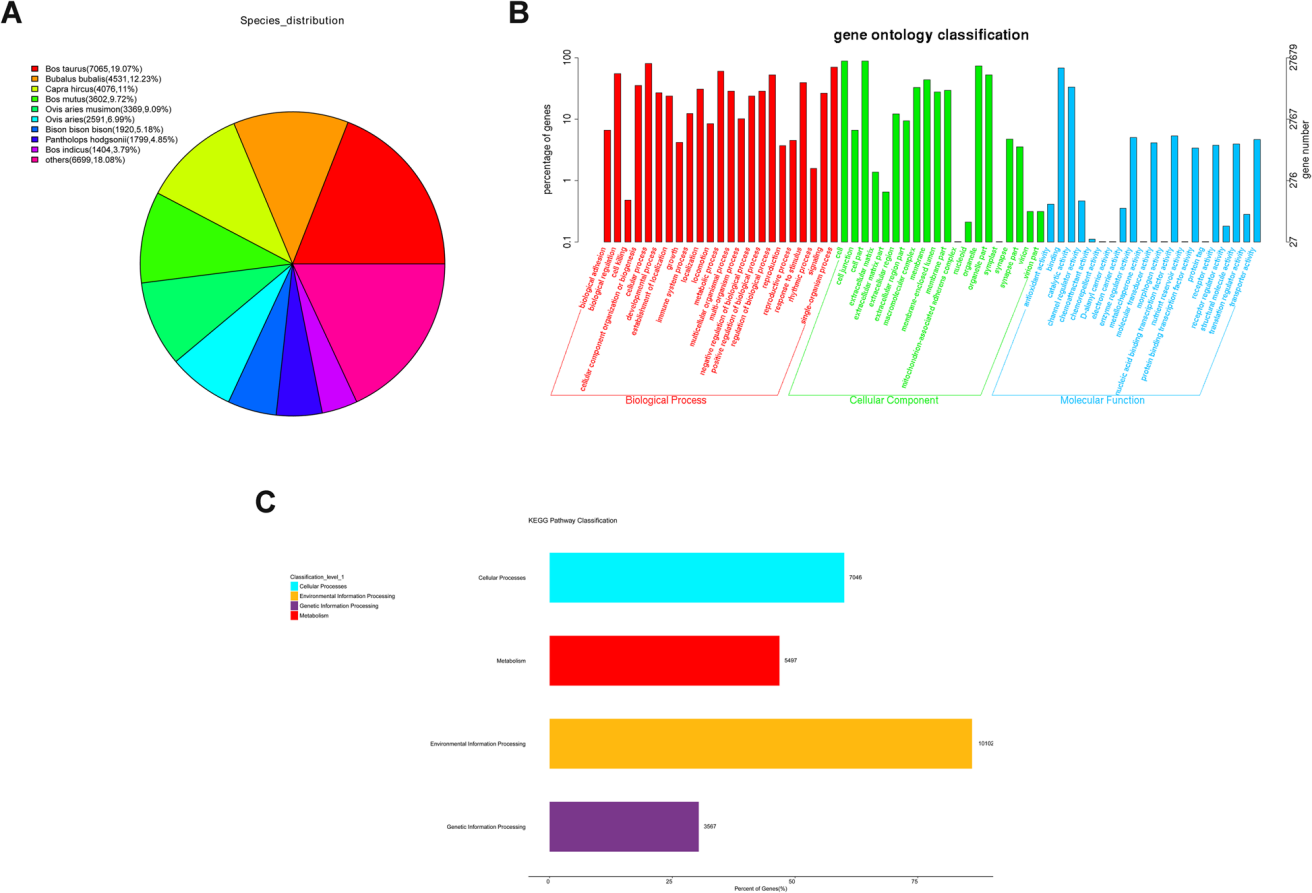
Species and functional annotations of unigenes. (**A**) Species distribution of unigenes annotated by the NR database. (**B**) GO annotation analysis for unigenes. (**C**) KEGG annotation analysis for unigenes

Although the reference genome of forest musk deer is currently available, the functional annotation information is relatively lacking. To obtain in-depth insight into the functions of the forest musk deer genes, the unigenes were annotated using the NCBI NR, SWISSPROT, KOG, KEGG, and GO databases. The results showed that a total of 40,851 unigenes were annotated by NR, SWISSPROT, KOG, KEGG, and GO databases. Among these unigenes, 37,056 (90.71%), 30,950 (75.76%), 24,836 (60.80%), 11,740 (28.74%), and27679 (67.76%) were mapped to the NR, SWISSPROT, KOG, KEGG, and GO databases, respectively (Table S2). NCBI NR alignment results showed that 19.07% of forest musk deer unigenes matched the genome sequences of *Bostaurus* (Fig. 3A). Additionally, 12.23%, 11%, 9.72%, and9.09% of the BLAST hits were identified within the reference protein databases of *Bubalus bubalis, Capra hircus, Bosmutus,* and *Ovis arie smusimon*, respectively (Fig. 3A). GO annotation analysis revealed that these unigenes were involved in the regulation of multiple essential biological processes such as immune system processes, cellular component organization or biogenesis, response to stimulus, growth, and cell killing (Fig. 3B). KEGG annotation analysis showed that these unigeness were divided into 4 categories (*i.e.* environmental information processing, cellular processes, metabolism, and genetic information processing) (Fig. 3C).

### Identification of DEGs and related GO/KEGG enrichment analysis

**Fig. 4:**
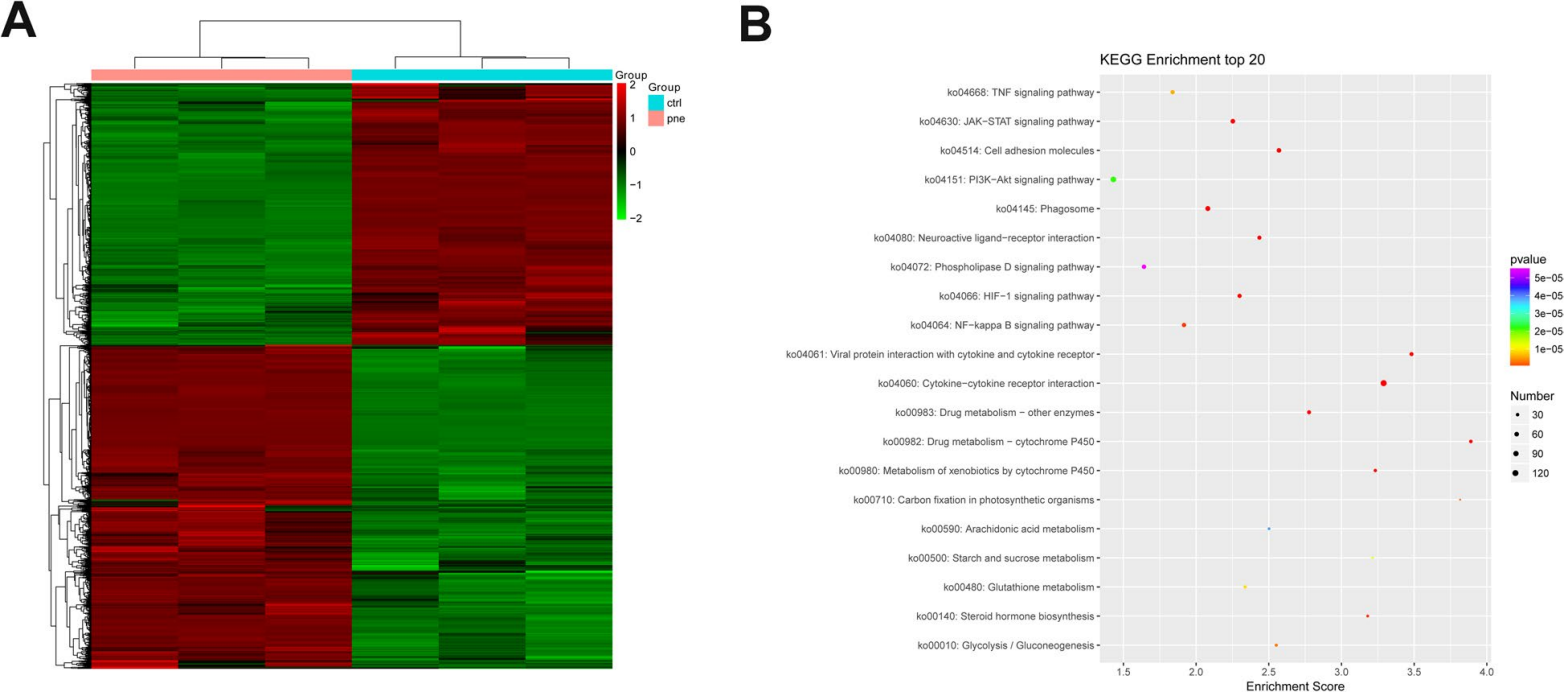
Identification of DEGs and related GO/KEGG enrichment analysis. (**A**) Heatmap of DEGsin diseased lung tissues of forest musk deer compared to the normal lung tissue group.The R package v3.6.3 and ComplexHeatmapR package v2.2.0 were used to create the heatmap. (**B**) Top 20 KEGG pathways enriched by DEGs [20]

Differential expression analysis revealed that 1805 unigenes were markedly up-regulated and 1460 unigenes were notably down-regulated in diseased lung tissues of forest musk deer compared to the normal lung tissue group (Table S3).The heatmap of these DEGs was shown in Fig. 4A. GO enrichment analysis showed that these DEGs were implicated in a host of biological processes including innate/adaptive immune responses, defense responses to pathogens, metabolism, and cell/tissue homeostasis (Table S4). Additionally, multiple KEGG pathways (*e.g.,* cytokine-cytokine receptor interaction, viral protein interaction with cytokine and cytokine receptor, cell adhesion molecules, JAK-STAT/TNF/NF-kappa B signaling pathways, and phagosome) were significantly enriched by these DEGs (Table S5), while these pathways have been well documented to be closely associated with innate and adaptive immune responses. The top 20 KEGG pathways are presented in Fig. 4B [20].

### Screening of DEGs related to lung injury, immunity, and pathogen infection

**Fig. 5:**
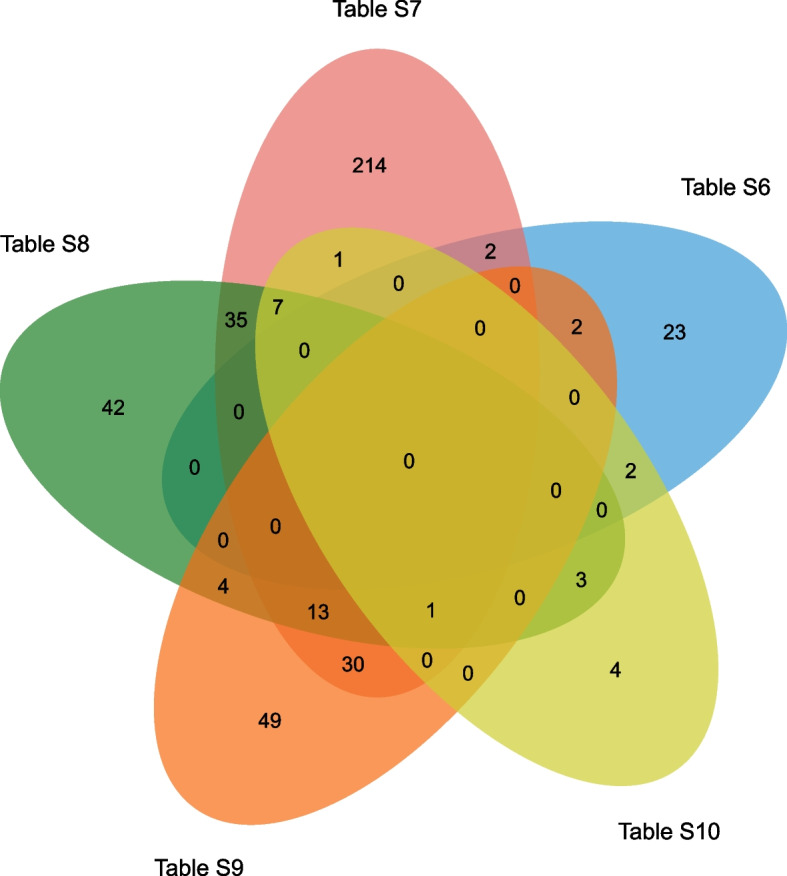
Venn analytical outcomes of unigene sets in Tables S6-S10. The upper section shows the Venn diagram of unigenes in Tables S6-S10. The middle histogram presents the number of unigenes in Tables S6-S10.The chart below displays the number of specific (1) elements of one list or shared elements by 2, 3, … lists. For instance, a total of 332 unigenes were specific to one list. In addition, 79 unigenes, 20unigenes, and 1 unigenewere shared by 2, 3, and 4 of the 5 lists, respectively

Next, DEGs related to lung development (Table S6), immunity (Table S7), or bacterial (Table S8)/viral (Table S9)/fungal (Table S10) infection were screened out from Table S4 by searching for items containing the keywords “lung”, “immune/immunoglobulin/immunity”, “bacterial/bacterium”, “virus/viral” or “fungiform/fungus/fungal”. The aberrant expression of these unigenes might be closely linked with the pathogenesis of ARDS in forest musk deer. Next, Venn analysis of the DEG sets in Tables S6-S10 was performed to identify the DEGs that might play vital roles in the above-mentioned two or more biological processes. The outcome of the Venn analysis is presented in Fig. 5 and Table S11. The results showed that 2, 2, 2 genes related to lung development might also be implicated in immunity (Table S6|Table S7), viral infection (Table S6|Table S9), and fungal infection (Table S6|Table S10). Additionally, 35 unigenes, 30 unigenes, and 1 unigenes were found to be involved in the immune responses to bacteria (Table S7|Table S8), viruses (Table S7|Table S9), and fungi (Table S7|Table S10), respectively. Moreover, 4 unigenes (TCONS_00011905, TCONS_00013874, TCONS_00032940, and TCONS_00030832) might be associated with infections in both bacteria and viruses (Table S8|Table S9). Additionally, 3 unigenes (TCONS_00016874, TCONS_00022939, and TCONS_00022940) related to both bacterial and fungal infections were identified (Table S8|Table S10). Additionally, there were 13 common genes in Tables S7-S9 (Table S7|Table S8|Table S9), which might play vital roles in the immune defenses against both bacteria and viruses. Additionally, 7 common unigenes were identified in Table S7, S8, and S10 (Table S7|Table S8|Table S10). These unigenes (Table S7|Table S8|Table S10) might be vital players in the immune responses to both bacteria and fungi. Furthermore, there was 1 common unigenes (TCONS_00028682) in Tables S7-S10 (Table S7|Table S8|Table S9|Table S10), suggesting that TCONS_00028682 (lactotransferrin) might be involved in the immune responses to bacteria, viruses, and fungi.

## Discussion

In this study, our RNA-seq analysis showed that 3265 unigenes were differentially expressed (1805 up-regulated, 1460 down-regulated) in lung tissues of forest musk deer with ARDS relative to the non-ARDS group. Moreover, we identified 29, 303,105, 99, and 18 DEGs that were potentially associated with lung development (Table S6), immunity (Table S7), bacterial infection (Table S8), viral infection (Table S9), and fungal infection (Table S10), respectively. Moreover, we further filtered out 2(TCONS_00023532 and TCONS_00023537), 2(TCONS_00040651, and TCONS_00040652), and 2(TCONS_00013967 and TCONS_00013968) DEGs that might play vital roles in lung injury related to immune system dysfunction, viral infection, or fungal infection, respectively. Among these genes, sonic hedgehog (SHH, corresponding unigenes IDs: TCONS_00013967 and TCONS_00013968) has been implicated in immunity, acute lung injury, and fungal infection. For instance, Karnam et al*.* demonstrated that fungi could activate the SHH signaling pathway, while SHH downstream genes (5/15-lipoxygenases (LO) or cyclooxygenase (COX)-2) could inhibit or promote proinflammatory cytokine production [24]. Chen et al*.* demonstrated that the inhibition of SHH signaling by its inhibitor cyclopamine could aggravate lung tissue damage and increase the thickness of alveolar septa in lipopolysaccharide (LPS, Gram-negative bacterium cell wall component)-induced acute lung injury mice [25]. Our RNA-seq data showed that SHH expression was notably down-regulated in diseased lung tissues of forest musk deer with ARDS compared with the normal group. These data suggested the close association of aberrant SHH expression and ARDS-related lung injury in forest musk deer.

Moreover, we identified 13 DEGs (e.g.,NLRC5, NLRP3 (NACHT, LRR and PYD domains-containing protein 3), tyrosine-protein kinase FGR, B-cell leukemia-3 (Bcl-3), neutrophil gelatinase-associated lipocalin (NGAL), and secretory leukocyte protease inhibitor (SLPI) that might play key roles in the immune defenses against both bacteria and viruses (Table S7|Table S8|Table S9). NLRC5 and NLRP3, members of the nucleotide-binding domain leucine-rich repeat-containing (NLR) family, have been identified as crucial players in inflammation, immunity, and host defenses against viruses and bacteria [26–28]. Moreover, prior studies showed that NLRP3 deficiency could markedly alleviate lung injury, ameliorate lung vascular permeability, reduce neutrophil infiltration into the lung, and inhibit pro-inflammatory cytokine secretion in an LPS-induced ALI mouse model [29]. Previous reports also pointed out that the overexpression of tyrosine-protein kinase FGR could weaken host defenses against viruses, bacteria, and parasites [30–32]. Bcl-3, a member of the IκB (inhibitor of NF-κB) family, functions as a vital player in immunity and NF-κB activity regulation [33, 34]. Kreisel et al*.* showed that Bcl-3 could attenuate acute inflammatory lung injury in mice [35]. NGAL plays a central role inantimicrobial immune responses, cell growth, metastasis, apoptosis, and differentiation [36–38]. Moreover, lipocalin 2 (Lcn2) (the mouse homolog of human NGAL) expression has been found to be markedly increased in septic mice with acute lung injury compared to those without acute lung injury [39]. SLPI, one of the first lines of defense against multiple bacteria (*e.g.,E. coli, Staphylococcus aureus, Mycobacterium bovis, Staphylococcus epidermidis, Mycobacterium tuberculosis,* and *Pseudomonas aeruginosa*) and fungi (*e.g., Aspergillus fumigatus* and *Candida albicans*), can protect the host from excessive inflammatory responses/tissue injury and initiate tissue/cell repair/regenerative processes [40]. Additionally, some studies revealed that SLPI plays a crucial role in protecting lung tissues from inflammatory and proteolytic damage and microbial infection [41, 42]. These data further suggested the vital roles of these genes in the immune defenses against pathogens.

Moreover, GO analysis suggested that tyrosine-protein kinase SYK, peptidoglycan recognition protein 1 (PGRP1), cathelicidin-6, cathelicidin-4, and chromogranin-A might serve as crucial players in the immune responses to both bacteria and fungi. Spleen tyrosine kinase (Syk) is involved in multiple biological processes such as innate and adaptive immunity, cellular adhesion, and vascular development [43, 44]. Moreover, previous studies showed that the inhibition of Syk reduced airway inflammation and facilitated mouse survival in an LPS-ALI mouse model [45].The repeated challenge of the fungal pathogen Aspergillus fumigates or β-glucan (the main cell wall component of various fungi) could lead to serious lung injury and the inhibition of Syk ameliorated β-glucan-induced inflammation, Th17/Treg imbalance, and lung tissue damage in BALB/C mice [46]. Peptidoglycan recognition protein 1 (PGRP1) belongs to the family of peptidoglycan recognition proteins, which not only have microbicidal activities against Gram-positive and Gram-negative bacteria and fungi but can also participate in immune and inflammatory responses [47–49]. Cathelicidins are a major family of host defense peptides that function as crucial players in inflammatory and immune responses and defenses against infections by pathogens including viruses, Gram-positive and Gram-negative bacteria, and fungi [50, 51]. Moreover, the expression levels of cathelicidin-3L2,cathelicidin-5,cathelicidin-6, cathelicidin-7, and cathelicidin-9 were notably increased in lung tissues of forest musk deer who died of purulent diseases compared with healthy individuals, while cathelicidin-4 was expressed at low levels in lung tissues of purulent individuals [52]. Our RNA-seq data showed that cathelicidin-3, cathelicidin-4, cathelicidin-5, cathelicidin-6, and cathelicidin-7 were all highly expressed in lung tissues of forest musk deer that died of ARDS, suggesting the close association of these cathelicidin family genes with lung injury and immune responses to bacteria and fungi. Lactotransferrin (lactoferrin), a glycoprotein widely present in most body fluids of mammals, possesses multiple pharmacological properties such as antiviral, antiparasitic, antifungal, anti-inflammatory, antimicrobial,and immunity-regulatory effects [53, 54]. Consistent with these data, we found that lactotransferrin was closely linked with immunity and pathogen (bacterium, virus, and fungus) infection.

Taken together, 3265 DEGs were identified in diseased lung tissues of forest musk deer that died of ARDS compared with normal lung tissues. This is the first study to disclose the lung gene alterations caused by ARDS in forest musk deer. Moreover, a total of 6 potential unigenes related to lung damage caused by immune abnormality (Table S6|Table S7) or viral (Table S6|Table S9)/fungal (Table S6|Table S10) infection were identified, which might contribute to the better management of lung injury induced by immune system dysfunction or pathogen (virus/fungus) infection. Additionally, we identified some unigenes that might play vital roles in the immune responses to bacteria (Table S7|Table S8), viruses (Table S7|Table S9), or fungi (Table S7|Table S10). Additionally, multiple unigenes that were involved in immune defenses against coinfection with multiple types of pathogens (bacteria + viruses: Table S7|Table S8|Table S9; bacteria + fungi: Table S7|Table S8|Table S10; bacteria + viruses + fungi: Table S7|Table S8|Table S9|Table S10) were screened out. Furthermore, DEGs in response to the single infection or dual infection of pathogens (bacteria, viruses, or fungi) were filtered out. We supposed that these unigenes might have potential value in the elimination of pathogens and improvement of resistance to bacterial/viral/fungal infection in forest musk deer. Moreover, our study might contribute to the better management of lethal acute respiratory diseases in forest musk deer. Both our data and previous studies showed that pathogen infection and immune dysregulation can trigger lung injury and result in forest musk deer death. Thus, it is imperative to maintain good breeding conditions and improve the immunity of forest musk deer. Treatment of an appropriate amount of immune enhancer might improve the immune and defensive abilities of forest musk deer against pathogens and benefit the long-term development of the forest musk deer breeding system.

## Supplementary Information

**Additional file 1.**

## Data Availability

The raw data of RNA-seq has been deposited to the GEO (https://www.ncbi.nlm.nih.gov/gds/?term =) with the number of GSE191259. The data that support the findings of this study are available from the corresponding author upon reasonable request.
